# Social and ecological factors influencing offspring survival in wild macaques

**DOI:** 10.1093/beheco/aru099

**Published:** 2014-06-17

**Authors:** Daphne Kerhoas, Dyah Perwitasari-Farajallah, Muhammad Agil, Anja Widdig, Antje Engelhardt

**Affiliations:** ^a^Jr. Research Group ‘Primate Sexual Selection’, German Primate Center, Kellnerweg 4, 37077 Goettingen, Germany,; ^b^Jr. Research Group ‘Primate Kin Selection’, Max-Planck Institute for Evolutionary Anthropology, Deutscher Platz 6, 04103 Leipzig, Germany,; ^c^Institute of Biology, University of Leipzig, Talstrasse 33, 04103 Leipzig, Germany,; ^d^Primate Research Centre, Bogor Agricultural University, Jl. Lodaya II/5, 16151 Bogor, Indonesia,; ^e^Faculty of Mathematics and Natural Sciences, Bogor Agricultural University, Kampus IPB Darmaga, Jl. Raya Darmaga, 16680 Bogor, Indonesia,; ^f^Faculty of Veterinary Medicine, Bogor Agricultural University, Kampus IPB Darmaga, Jl. Raya Darmaga, 16680 Bogor, Indonesia, and; ^g^CRC ‘Evolution of Social Behaviour’, Georg-August University, Kellnerweg 6, 37077 Goettingen, Germany

**Keywords:** between-group encounters, female reproductive success, Macaca nigra, offspring loss, proportional hazards model, socioecology.

## Abstract

Premature loss of offspring decreases direct fitness of parents. In gregarious mammals, both ecological and social variables impact offspring survival and may interact with each other in this regard. Although a number of studies have investigated factors influencing offspring loss in mammals, we still know very little on how different factors interact with one another. We therefore investigated fetal and infant mortality in 3 large groups of wild crested macaques (*Macaca nigra*) over a period of up to 5 years by including potential social causes such as maternal dominance rank, male immigration, between group encounters, and ecological conditions such as rainfall in a multivariate survival analysis using Cox proportional hazards model. Infant but not fetal survival was most impaired after a recent takeover of the alpha-male position by an immigrant male. Furthermore, infant survival probability increased when there was an increase in number of group adult females and rainfall. Fetal survival probability also increased with an increase of these 2 factors, but more in high-ranking than low-ranking females. Fetal survival, unlike that of infants, was also improved by an increase of intergroup encounter rates. Our study thus stresses the importance of survival analyses using a multivariate approach and encompassing more than a single offspring stage to investigate the determinants of female direct fitness. We further provide evidence for fitness costs and benefits of group living, possibly deriving from high pressures of both within- and between-group competition, in a wild primate population.

## Lay summary

Better to live in a big group if you want your offspring to survive! Using a multivariate approach, we show how the interplay of ecological and social factors influences fetus and infant survival in wild crested macaques. Offspring are more likely to survive in bigger groups, but seasonality also influences their survival. Fetus survival is higher for higher ranking mothers, whereas the main determinant of infants’ death is an alpha-male takeover by an immigrant male.

## INTRODUCTION

Direct fitness is defined as the number of adult offspring left by an individual in the next generation (Williams 1996). Premature loss of offspring thus limits parents’ reproductive success and creates costs in terms of investment already made in progeny (Trivers 1974). There is an extensive body of literature on the factors impacting female reproduction, specifically offspring survival, in mammals (Bronson 1985, 1989; Clutton-Brock 1988) both under captive (e.g., Lim and Wang 2010; Kavanagh et al. 2011) and natural conditions (e.g., rodents: Larsen and Boutin 1994; carnivores: Bertram 1975; Frank et al. 1995; Wahaj et al. 2007; Watts and Holekamp 2008, 2009; ungulates: Pluháček and Bartoš 2005; primates: Roberts et al. 2012). However, we still know little of the influence of the interaction of social and ecological factors on offspring loss. Furthermore, most studies focused solely on a specific offspring stage, either pre- or postnatal, although the determinants of offspring death may vary between these stages. Events occurring in the prenatal stage, such as the causes of miscarriages, have either not been reported or little investigated under natural conditions, typically due to the difficulty of monitoring spontaneous abortions (but see Beehner, Nguyen, et al. 2006; Beehner, Onderdonk, et al. 2006). More comprehensive studies are therefore needed to better understand the interactions between social and ecological factors causing offspring loss in wild female mammals.

From the data available, it is clear that a number of parameters can impact offspring survival. In addition to maternal, paternal, or offspring properties (e.g., female parity: Pluháček et al. 2007; female age: Packer et al. 1998; female dominance rank: Majolo et al. 2012; offspring genetic abnormalities: Wilmut et al. 1986; reviewed in Pusey 2012) especially influencing fetal loss in the first trimester of pregnancies, both ecological and social variables have been shown to influence offspring (i.e., fetus and infant) survival. One of the most important is climate, as its components such as rainfall and temperature have great impact on plant productivity (e.g., Terborgh and Janson 1986) and thus food availability (Kim et al. 2012). Because female fecundity as well as offspring survival depend highly on food availability, climatic variables can modify female reproductive success (e.g., ungulates: Kruuk et al. 1999; carnivores: Russell et al. 2002; bats: Grindal et al. 1992; primates: Dittus 1977; Altmann and Alberts 2003; Beehner, Nguyen, et al. 2006). Additionally, in gregarious species, social factors act through the influence of other group members and rival groups. Female competition both within and between groups is an important selective force on females (e.g., rodents: Schradin et al. 2010; carnivores: Clutton-Brock et al. 2006; primates: Sterck et al. 1997) although only few studies have addressed the reproductive consequences of between-group competition. However, females and their offspring also benefit from living with conspecifics, for example, through enhanced allomaternal care and joint resource defense against other groups, as well as protection against predators and infanticide; hence, female social bonds and support can be important determinants of female reproductive success (reviewed for mammals: Silk 2007a and primates: Silk 2007b).

Additionally, males can have a positive effect on offspring survival by providing paternal care (e.g., some carnivores: Clutton-Brock et al. 2001 and primates: Goldizen and Terborgh 1989; Charpentier et al. 2008; Huchard et al. 2013; Langos et al. 2013). Males are, however, also well known to cause offspring death, particularly in species in which infanticide is a sexually selected male strategy (van Schaik and Janson 2000). They may even harm offspring before birth by increasing stress in pregnant females, particularly when newly immigrating males takeover the alpha position (e.g., equids: Pluháček and Bartoš 2005; rodents: Labov 1981; primates: Engh et al. 2006; Roberts et al. 2012).

Little is known to date about the relative importance of interaction between the aforementioned ecological and social factors in impairing offspring survival although it is clear that several of these potentially interact with each other, for example, the effects of food availability and group size (Ebensperger et al. 2012). Only 2 studies carried out on baboons have so far used multivariate analysis to investigate the determinants of offspring survival (Cheney et al. 2004; Beehner, Nguyen, et al. 2006). Both found that offspring loss is influenced by both ecological conditions and social factors. However, these studies as others on the determinants of survival usually focus on a single offspring stage (either pre- or postnatal, e.g., Wilmut et al. 1986; Packer et al. 1998; Pluháček et al. 2007; Majolo et al. 2012; Roberts et al. 2012).

The aim of our study was, therefore, to investigate simultaneously the combined effect of social and ecological factors, using modern statistical tools, on pre- and postnatal offspring survival in a wild population of crested macaques (*Macaca nigra*), living on the island of Sulawesi. In this species, miscarriages have been observed in the wild, and infant mortality reaches 20% (Engelhardt and Perwitasari-Farajallah 2008), although predation pressure is thought to be low due to the absence of large felids on Sulawesi (van Schaik 1989).

Crested macaques are categorized as one of the most socially tolerant species within the macaque genus, in which females form strong social bonds in extended social networks (Thierry et al. 2000). We thus expect all offspring to benefit from an increased number of adult female group members due to improved chances of successful resource access and monopolization through defense against adjacent groups. In addition, female social rank seems to be of little importance in females of this species with low-intensity conflicts and moderate dominance asymmetry (Duboscq et al. 2013). Hence, we predict maternal rank to have relatively little effect on offspring survival probability. At the same time, intergroup encounters should be stressful for females, given that, in wild crested macaques, intergroup encounters in which females are involved are often aggressive (Kinnaird and O’Brien 2000) and injuries inflected to females are observed on a regular basis. In fact, between-group aggression is predicted to be high in this species (van Schaik 1989; Sterck et al. 1997). We thus predict spontaneous abortion to be more likely with an increasing rate of stress through more frequent intergroup encounters. Finally, the high turnover rate by immigrants males may lead to frequent instability in the adult male dominance hierarchy (Neumann et al. 2011). Given that these instabilities may lead to increased tension within the group, we expect abortion to be more likely with an increasing rate of male immigration and changes in the male hierarchy. Dependent infants, on the other hand, should suffer specifically from takeovers of the alpha-male position by newly immigrating males given the potential risk of infanticide under these circumstances. We test our predictions with a Cox proportional hazards model in 3 groups over a study period of a total of 152 group months.

## METHODS

### Study site and subjects

Data were collected within the Macaca Nigra Project on 3 groups of wild crested macaques (R1, R2, and PB) living in the Tangkoko-Duasudara Reserve in Sulawesi, Indonesia (1°31′00.1″N, 125°10′59.9″E). This reserve, composed of primary and secondary lowland rainforest, covers an area of 8867 ha and ranges from sea level to 1350 m (O’Brien and Kinnaird 1997; Rosenbaum et al. 1998; Whitten et al. 2001). Temperatures are relatively constant throughout the year, with a monthly mean minimum and maximum of 23 and 28 °C, respectively. Annual rainfall ranged between 1410 and 2352mm during the study period (mean of 1940mm per year).

The study groups were fully habituated to human observers (Duboscq et al. 2008), and adults and infants were individually recognized. Group sizes varied from 50 to 80 individuals and included 4–11 adult males and 13–25 adult females (Supplementary Table S1).

Female crested macaques in this population give birth year round, but more than 80% of births occur within 5 consecutive months, namely January to May and up to 59.3% within 3 consecutive months, namely March to May (Engelhardt and Perwitasari-Farajallah 2008). As is the case in other macaques (e.g., Tanaka 1992), infant weaning started at 5 months of age (average of first nipple deterrence observation at 154.57 days of age, behavior described in Thierry et al. 2000) and was usually completed when the infant was approximately 1-year old.

### Data collection

Observations on groups R1 and R2 took place from March 2006 to December 2010 and for group PB from January 2008 to December 2010. Each of the 3 groups was followed at least once a week but usually several times per week from dawn to dusk (3894 total group observation days across the 3 groups, i.e., 75.5% days of the whole observation period). We recorded migrations, births, and disappearance/death of individuals, as well as the occurrence and size of female sex skin swellings (a sign of monthly ovarian activity; Higham et al. 2012) that lasts on average 19.4 days (Engelhardt and Perwitasari-Farajallah 2008) and encounters between groups, using *all-occurrence* sampling (Altmann 1974). In addition, outcome of dyadic aggressive interactions and displacements was recorded, during focal sampling of adult males and females (data extracted from Engelhardt and Perwitasari-Farajallah 2008; Neumann et al. 2010, 2011; Duboscq et al. 2013) and ad libitum sampling (Altmann 1974), to determine dominance rank through Elo rating, a method robust to frequent hierarchy changes (Neumann et al. 2011). Females were considered adult after conceiving their first living infant and males were considered adults after their scrota descended and their canines erupted.

We collected data on 99 fetuses and 78 infants (126 offspring altogether from 60 females). Onset of pregnancy was determined through the cessation of regular swelling cycles (i.e., a female not displaying her monthly sexual swellings for more than 2 consecutive months), as sexual swellings in this species are a reliable signal of ovulation (Higham et al. 2012) and there is no postconception swellings in this species (Hadidian and Bernstein 1979), confirmed by subsequent delivery or miscarriage. Miscarriages were detected through the observation of massive hemorrhaging from the vagina (sometimes with a protuberant umbilical cord) followed by the resumption of sexual activity and sex skin swelling (after a mean of 26.5 days ± 20.2, *N* = 17). Menses is difficult to detect in wild adult female crested macaques. To ensure that vaginal bleeding truly derived from fetal loss and not from menses, we only counted those cases in which the female had not displayed any swelling during the previous 2 months, thus suggesting that she was pregnant. This means that we may have missed some early miscarriages occurring during the first 2 months of the pregnancy. Pregnancy with life birth in this species last on average 170 days (Thomson et al. 1992) and we observed in this population a range of pregnancies lasting from 171 to 185 days.

Infants were observed during their earliest life phase, that is, during the period in which infant mortality is highest in mammals (Caughley 1966). In macaques and other cercopithecine primates, this period covers the first year of life (e.g., van Noordwijk and van Schaik 1999). Accordingly, we recorded disappearances and deaths (from here on called “deaths” only) for individuals under the age of 1 year.

### Data analysis

We used fetal and infant survival as a binary response variable in 2 separate models. The period of fetal survival was considered from conception (the last day of sexual swelling of the last estrous cycle) to birth and the period of infant survival was considered as the date of birth until 1 year of age. Fetuses were scored as alive or dead for each of up to two 90-day intervals from the defined day of conception to miscarriage or birth and for infants up to four 90-day intervals from birth to death or 1 year of age (496 intervals in total) in order to incorporate time-dependent variables into the model (Perperoglou et al. 2006). Infants for which the day of conception was known (*N* = 71) occurred twice in the analyses: once as fetus and once as infant. Each quantitative variable was calculated on a daily basis and we used the mean over each 90-day intervals per individual. We considered the following predictor variables as potentially influencing fetal and/or infant survival:

• *Mean rainfall* (with an offset of 3 months) as an approximation of environment seasonality. It is well known that there is a link between phenology and water availability in seasonal tropical forests (van Schaik et al. 1993). Accordingly, increased rainfall leads to increased availability of fruits in the study area (Kinnaird and O’Brien 2000) and fruits are the major food source of crested macaques in Tangkoko (O’Brien and Kinnaird 1997). We shifted actual rainfall values back by 3 months, given that female crested macaques respond reproductively to environmental changes with a time lag of 3 months (Supplementary Figure S2).
• *Number of adult females* in the group as a measure of female–female within-group competition for resources (Isbell 1991) on one hand and of competitive ability during between-group competition on the other.
• *Maternal dominance rank* as a measure of individual competitive ability using Elo ratings based on aggressive dyadic interactions as well as displacements (Albers and de Vries 2001; Neumann et al. 2011). For each day, we standardized the Elo ratings of all adult females in each group (range of 0–1, 1 being the highest). Maternal rank is assumed to be transferred to the infant in macaques (Berman 1980).
• *Male hierarchy instability* as a measure of increased within-group aggression and stress. Male hierarchical instability was calculated using the weighted hierarchy instability index (cf. Neumann et al. 2011) based on all the adult males’ daily dominance rank assessed through Elo rating (as described above).
• *Number of male immigration* events as another measure of increased within-group aggression was scored as a binary measure “0” or “1” to balance the data set containing mainly no occurrence.
• *Alpha-male takeovers* by a newly immigrated male (*N* = 13 events) as yet another measure of increased within-group aggression and as a measure of the risk of infanticide. We measured the proportion of days a new alpha-male was present per interval until the fetus was born or the infant reached 6 months old, as some mother start cycling again by then.
• *Intergroup encounter* daily rate (i.e., 2 groups in visual range, *N* = 1126 events) as a measure of conflict resulting of between-group competition.
• We furthermore controlled for *fetus and infant age* and *infant sex*. Miscarried fetus sexes were unknown.

We incorporated interactions into both models. An interaction between 2 or more predictor variables indicates that the effect on the response of one of these predictor variables is conditional on the state or value of the other predictor variable and not simply additive (Quinn and Keough 2002). We added a 3-way interaction between rainfall (our measure of environmental seasonality), number of adult females in the group, and maternal dominance rank, as well as all lower order interactions between these particular variables to our model, because these variables are known to have an interdependent effect on resource access and thus offspring survival (Pusey et al. 1997; van Noordwijk and van Schaik 1999; King et al. 2005). The number of individuals within a group influences individual resource access through the degree of within-group competition as well as success of between-group resource defense (van Schaik 1989). Furthermore, fruits, being usually seasonal and patchily distributed, increase within-group contest competition, which again increases with the number of competitors. Finally, with increasing degree of within-group competition, high-ranking females get an advantage over low-ranking females in regard to resource access and reproduction. In addition, we incorporated 3 nested random factors into the model to control for the multiple source of random error: group identity, mother identity, and offspring identity.

### Statistical analysis

We used a Cox proportional hazards model (Therneau and Grambsch 2000) to test the influence of the predictor variables on fetal and infant survival, respectively. The Cox model is a nonparametric survival analysis (Pletcher 1999) that allows the influence of several parameters to be estimated simultaneously and over time on the risk of death (i.e., the hazard rate), whereas no assumption is made concerning the shape of the hazard function. In our Cox model, the regression coefficients represent the log change in the hazard function per unit increase of the predictor variable. In other words, a negative hazard rate (β) indicates increased survival chances with increasing value of the predictor variable (Therneau and Grambsch 2000). The model was fitted in R (version 2.14.0, Fox and Weisberg 2011) using a mixed effects model variant of the Cox model, namely the function “coxme” (package “coxme,” R Development Core Team 2011) in order to include the 3 nested random factors. We checked each predictor for its distribution; to obtain approximately symmetrical distributions, we square-rooted age and intergroup encounter rate and double square-rooted male hierarchy stability. Subsequently, all quantitative predictor variables were *z*-transformed to a mean of 0 and a standard deviation of 1. We determine the statistical significance of the 2 full models by comparing their fit with the respective null model (containing only the random and control factors) with a log-likelihood ratio test. The variance inflation factors derived from a linear model containing all the predictors except the random effects (function “vif” of the R package “car,” Quinn and Keough 2002; Fox and Weisberg 2011) revealed that collinearity was not an issue for both models (largest variance inflation factor is 2.1). The specific time interval of 90 days for a given offspring (i.e., fetus and infants) is the unit of analysis. When a fetus was aborted or an infant died, the time interval containing this event was included in the analysis. Variables may be fixed over time (e.g., infant sex) or time dependent (e.g., rainfall) (Beyersmann and Schumacher 2008). All the terms included in the models are specified in Tables 1 and 2, except for the 3 nested random factors.

**Table 1:** Cox mixed model results for fetus survival (*N* = 184)

Predictor	β	*z*	*P* value
Rainfall	−0.504		
Number of adult females	−1.751		
Maternal dominance rank	0.336		
Male immigration	0.100	0.23	0.820
Male hierarchy stability	−0.853	−1.43	0.150
Male takeover	−0.498	−0.64	0.520
**Intergroup encounter rate**	−**1.509**	−3.09	**0.002**
Fetal age	−0.027	−0.07	0.940
Rainfall × maternal dominance rank	1.009		
Number of adult females × maternal dominance rank	1.055		
Number of adult females × rainfall	−0.728		
**Rainfall × number of adult females × maternal dominance rank**	1.036	2.36	**0.018**

Significant effects are highlighted in boldface. β is the hazard rate coefficient; a positive value indicates an increased risk of dying with increasing value of the predictor. *z* and *P* values not shown are uninformative because the respective term is involved in a higher order interaction.

**Table 2:** Cox final reduced mixed model results for infant survival (*N* = 260)

Predictor	β	*z*	*P* value
Rainfall	−0.319		
Number of adult females	0.460		
Maternal dominance rank	−0.457	−1.35	0.180
Male immigration	−0.683	−1.58	0.110
Male hierarchy stability	0.092	0.23	0.820
**Male takeover**	**2.928**	3.22	**0.001**
Intergroup encounter rate	−0.031	−0.07	0.950
Infant age	−0.417	−1.18	0.240
Infant gender	−0.251	−0.39	0.700
Mother parity	−0.276	−0.83	0.410
**Number of adult females** × **rainfall**	0.697	2.00	**0.045**

Significant effects are highlighted in boldface. β is the hazard rate coefficient; a positive value indicates an increased risk of dying with increasing value of the predictor. *z* and *P* values not shown are uninformative because the respective term is involved in a higher order interaction.

## RESULTS

### General results

Overall, during the study, 18% of the 99 conceptions resulted in abortions and 17 of the 78 infants (22%) disappeared in their first year of life. We found the body of 8 of these 17 infants. All but one of these infant bodies showed large puncture wounds (Supplementary Table S3).

### Models

#### Fetal survival

**Figure 1:**
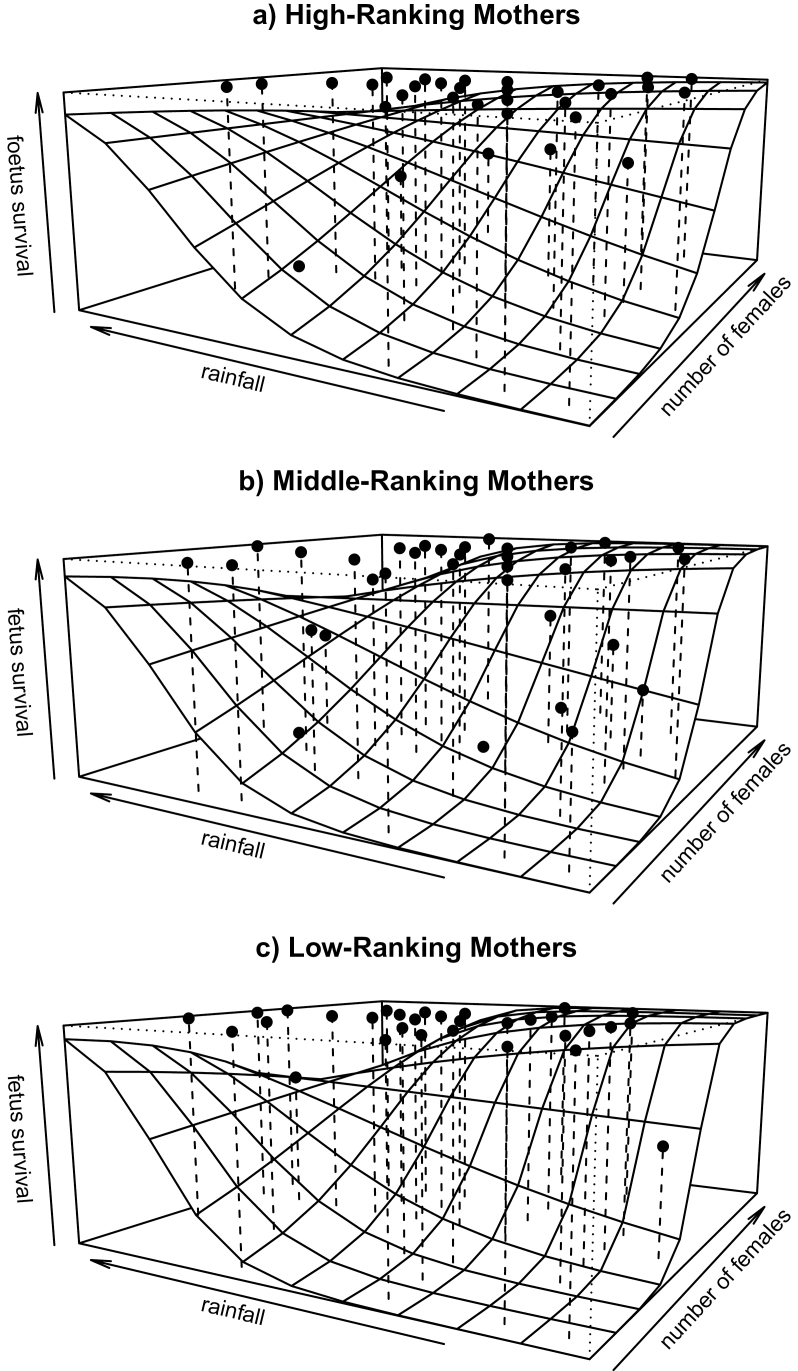
Effect of rainfall and number of group adult females on the survival likelihood of fetuses of high-ranking (a), middle-ranking (b), and low-ranking (c) mothers. The continuous variable dominance rank was divided into 3 categories (high, middle, and low) to enable plotting. The plane depicts values predicted by the fetus Cox mixed model with each grid representing the mean value per square of the predicted mixed model. Dots represent mean empirical survival rates value per square.

Overall, the model revealed that the set of predictor variables used had a clear influence on the probability of fetal survival (integrated log-likelihood ratio test comparing the fit of the full model with the fit of a null model containing only the random effects; χ^2^ = 24.32, degrees of freedom [df] = 11, *P* = 0.01). The model results indicate that the rate at which a group encountered a neighboring group had the strongest effect on fetal survival (Table 1): the more frequent intergroup encounters were, the more likely those fetuses survived. In addition, the 3-way interaction between rainfall, number of adult females in the group, and maternal dominance rank had a significant impact on fetal survival (Table 1). In general, fetuses were more likely to survive with an increase in rainfall and the greater the number of females present in the group (Figure 1a–c). There were, however, differences between female rank classes in terms of when and to what extent these positive effects set in. Fetuses of high-ranking females benefitted most and constantly from an increase in rainfall compared with fetuses of middle- and low-ranking females. Fetuses of middle- and low-ranking females, in contrast, only benefitted from an increase in the number of group females when rainfall was low to moderate. When both female number and rainfall were high, fetuses of high-ranking females were most likely and those of low-ranking females least likely to survive. Alpha-male takeover rate, occurrence of male immigration, male hierarchy stability, and fetal age did not have any significant impact on fetal survival (Table 1).

#### Infant survival

The model revealed that, overall, the full set of predictor variables used did not explain the variation in infant survival (integrated log-likelihood ratio test comparing the fit of the full model with the fit of a null model containing only the random effects; χ^2^ = 20.49, df = 14, *P* = 0.11). However, because the 3-way interaction between rainfall, number of adult females in group, and maternal dominance rank did not reveal significance (β = 0.06, *z* = 0.19, *P* = 0.85) and two of the constituent 2-way interactions were also not significant (maternal dominance rank × rainfall: β = −0.11, *z* = −0.31, *P* = 0.75; maternal dominance rank × number of females in group: β = −0.21, *z* = −0.69, *P* = 0.49; determined from a model not comprising the 3-way interaction), we excluded these interactions from the full model. Although there is a risk of multiple testing (Mundry and Nunn 2009), exclusion was conducted successively (however, we did not use stepwise deletion) to ensure that none of the potentially significant interactions remained undetected.

**Figure 2:**
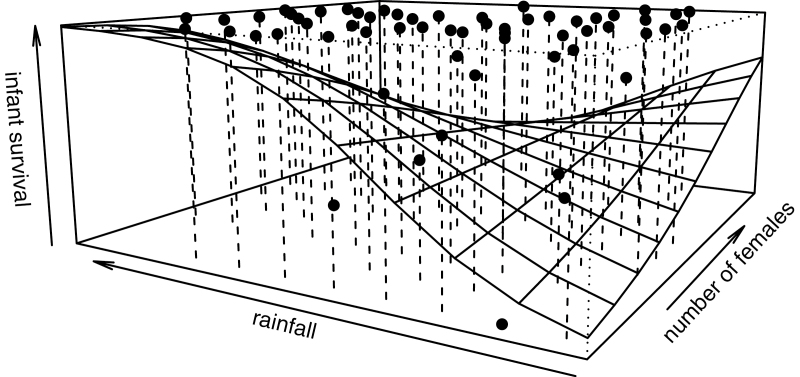
Effect of rainfall and number of group adult females on the likelihood of infant survival. The plane depicts values predicted by the infant Cox mixed model with each grid representing the mean value per square of the predicted mixed model. Dots represent mean empirical survival rates per square.

The results of the final reduced model showed that all single parameters and the remaining significant 2-way interaction (number of females × rainfall) explained the variation in infant survival (integrated log-likelihood ratio test: χ^2^ = 21.83, df = 11, *P* = 0.02). According to this model, infant survival probability was most strongly affected by takeover of the alpha-male position (Table 2). Such a takeover almost tripled the probability of infants dying (β = 2.9). Interestingly, instability in the male hierarchy per se did not have such an effect nor did the immigration of males into a group. Rainfall and the number of adult females per group also significantly influenced the probability of infant survival (Table 2); infants were more likely to survive with increasing rainfall and the greater the number of females in the group (Figure 2). From a certain degree of rainfall and number of females onward, however, the positive effect of each of these variables was weakened, as in the fetus model. Infant age and infant sex did not have any significant effect on survival probability.

## DISCUSSION

Our results strongly indicate that an interplay of social and environmental factors significantly influenced fetal and infant loss in wild crested macaques. Our results also showed that the determinants of survival may differ between offspring stages. In both models, a social variable best explained variation in survival, but the precise variable differed between fetuses and infants. Similarly, in both models, the interaction between an ecological and a social factor exerted a significant effect on offspring survival, but again, our analyses revealed differences in the details of survival determinants between different offspring stages. Our results thus stress the importance of investigations covering more than a single offspring stage.

For both offspring stages, a social variable best explained variation in survival. Fetuses had a greater survival chance the more often their group was involved in intergroup encounters, whereas infants had a reduced survival chance after a new male had taken over the alpha-male position. Our finding that fetuses are more likely to survive the more often their group is involved in intergroup encounters is surprising to us. We had expected the opposite to be the case because aggressive intergroup encounters may increase female stress levels (Pride 2005) and thus may cause pregnancy failures (reviewed in Nakamura et al. 2008). In addition, between-group aggressions have been predicted to be important in crested macaques for female social interactions (van Schaik 1989; Sterck et al. 1997), as between-group competition may have an impact on resource access, hence on female dominance interaction and reproduction (Kinnaird 1994). Nevertheless, our data indicate that intergroup encounters do not impair but foster female pregnancies, a finding that is difficult to explain through direct advantages. One possible explanation may be that the rate with which a group comes into conflict with neighboring groups correlates with the quality of this group’s home range. It has, for example, been shown for Japanese macaques that in areas with food of higher quality, intergroup encounters occur more often than in areas with food of lower quality (Sugiura et al. 2000). Similarly, in our study population, multiple groups compete over fruit trees and the frequency of aggression during intergroup encounters increases when fruit becomes scarce and defensible (Kinnaird and O’Brien 2000). Improved fetal survival may thus simply reflect preferential access to food by these groups during food shortages. Future studies might therefore incorporate the influence of winning or losing intergroup encounter and test the prediction that winning an intergroup encounter increases fetal survival.

The assumption that between-group competition and environmental seasonality play a role in fetal survival in our study population is also supported by our finding that an increase in both the number of group adult females and rainfall in principle improved fetal survival probability. Groups with more adult females are better able to defend resources against other groups (Sterck et al. 1997). Rainfall, at the same time, may lead to an increase in fruit availability in the study area (Kinnaird and O’Brien 2000) and as such to an increase in the most important food source of crested macaques (O’Brien and Kinnaird 1997). As a consequence, pregnant females can better cope with the energetic costs of pregnancy (Small 1982). Our findings thus suggest that in terms of successful pregnancy, improved access to food outweighs the costs of stress received during intergroup encounters in crested macaques. Interestingly, there seems to be a limit to which this is the case. Our model shows that from a certain group size and degree of rainfall (i.e., environmental seasonality) onward, the positive effect is diminished in the fetuses of middle-ranking and even more so in those of low-ranking females. Fruits, particularly when occurring clumped in large trees (as is the case in Tangkoko, Kinnaird and O’Brien 2000), are highly defendable food sources that increase within-group contest competition (Mathy and Isbell 2001). Within-group contests also increase with the number of females competing for clumped food (van Schaik 1989). Our results thus suggest that under certain conditions (patchily distributed food and many competitors), the costs of within-group competition override the positive effect of group resource defense for middle-ranking and lower ranking females. This may explain why dominance hierarchies remain in female crested macaques (Duboscq et al. 2013) although the species is relatively less despotic than other macaque species (Sueur et al. 2011; Micheletta et al. 2012; Micheletta and Waller 2012; Duboscq et al. 2013). When food is less clumped, on the contrary, competition between females of the same groups seems to be less detrimental and/or balanced by the positive effect of joint resource monopolization. The reason for this is most likely that females feed more on dispersed and less defendable food sources during such periods (Kinnaird and O’Brien 2000) and thus engage in fewer direct contests.

Based on the reduced model, between-group competition and seasonality also seem to influence infant survival likelihood in our study population. As in fetuses, infants in general benefit from an increase in rainfall and in the number of adult females in the group, but their survival likelihood then decreases when both parameters simultaneously increase beyond a certain point. However, in contrast to fetuses, there is no rank-specific difference, suggesting that all infants suffer similarly from high levels of within-group competition. Why the infants of high-ranking females (in contrast to their pregnant mothers) should suffer equally from within-group contest remains unclear. Most likely, the infants of high-ranking females are not yet able to successfully deploy their status (compare Datta 1988) during food competition. Macaque infants, as in several other Old World monkeys, do not hold a position within a dominance hierarchy during their first months of life and depend on support from others during agonistic encounters (Berman 1980; Pereira 1989).

Environmental seasonality and female group size were nevertheless not the most important determinants of infant survival in our study population. More important was whether or not the alpha-male position was taken over by a recent immigrant after the infant had been sired. In cases where a newly immigrating adult male reached alpha-rank position after an infant had been conceived, infants were 3 times more likely to die. We do not have direct evidence of these males having killed the respective infants—although males have been observed to attack infants—but the resemblance to cases of infanticide conducted as a male reproductive strategy is striking (e.g., Hrdy 1974; Hausfater 1984; van Schaik and Janson 2000; Pluháček and Bartoš 2005). In many of the cases in which we were able to retrieve the body, these exhibited deep punctures in the head and thorax consistent with having been inflicted by adult male canines, as it has been observed in other macaque species (cf. Ciani 1984; De Ruiter et al. 1994; Soltis et al. 2000). Because there are no large felids on the island of Sulawesi, we feel justified in assuming these wounds stem from male bites. Interestingly, general immigration by males and instability in the dominance hierarchy did not have a significant effect on infant survival probability. This suggests that infants are not more likely to die as a byproduct of increased aggression between males in general (as suggested, e.g., in Bartlett et al. 1993) but that infant death is specifically related to the arrival of a new alpha-male. It may well be that infanticide is a male reproductive strategy in crested macaques, given that dominant males appear to be able to monopolize access to females during the period of likely conception (Higham et al. 2012) and that new alpha-males in the vast majority of cases originate from other groups and are thus very unlikely to have fathered the infant killed (13 out of 14 takeovers during our study period).

Interestingly, several parameters previously found to impact fetal and infant survival in several species did not show any effect on offspring survival in our study. Fetal age usually has an effect on survival probability with younger fetuses (e.g., Beehner, Nguyen, et al. 2006) being more likely to die. Given that in humans the majority of miscarriages occur in the first trimester of pregnancy (e.g., Wilmut et al. 1986), we may have missed such an age effect because we were only able to detect miscarriages from the second trimester onward. The majority of early miscarriages, however, results from genetic aberrations (Ljunger et al. 2005), which were not the focus of our study.

We also did not find any significant effect of infant age or sex or maternal parity on infant survival probability although all three have previously been reported in mammals (e.g., parity: infant mortality is higher for primiparous females, Pluháček et al. 2007; infant age: younger infants die more than older infants, Altmann et al. 1977; infant sex: higher mortality among male than female infants, Cheney et al. 2004). The reason for our result is possibly that in our study population, infants seem to die mainly from infanticide and that males perform infanticide opportunistically, that is, without regard to infant sex or age (as long as the infant is still dependent) or the mother’s parity. Furthermore, reports of sex differences in infant mortality are inconsistent for primates (Clutton-Brock 1991) and sex-dependent mortality seems not to apply to macaques during the first year of life (van Noordwijk and van Schaik 1999). Similarly, the impact of parity on infant survival seems to be insignificant for most primates (van Noordwijk and van Schaik 1999; Cheney et al. 2004; but see Schino and Troisi 2005). Other more important variables (such as the number of adult females, rainfall, and alpha-male takeover) may thus have overridden any such effect in our statistical analysis. In addition, these variables may not play a role at such an early stage; thus, future studies should include older offspring stage too.

In summary, our study suggests that a combination of ecological constraints and social variables, in our case environmental seasonality, female within-group competition for food sources, between-group resource defense and male reproductive strategies, and their interactions, have an impact on offspring survival in crested macaques. It therefore reveals the direct link between fitness costs and benefits of sociality in regard to within- and between-group competition. We further show that the determinants of direct fitness have to be measured at both pre- and postnatal levels because they cannot necessarily be extrapolated from one to the other. Future studies adopting a similar approach would further elucidate the complex interplay of social and environmental determinants of direct fitness in gregarious mammals.

## SUPPLEMENTARY MATERIAL

Supplementary material can be found at http://www.beheco.oxfordjournals.org/

## FUNDING

This work was supported by the German Research Council (EN719/1,2 to A.E., WI1808/3-1 to A.W.), the Federal Ministry for Economic Cooperation and Development (EN719/1 to A.E.), the German Academic Exchange Service (DAAD), the Leakey Foundation, Primate Conservation Inc., and the International Primatological Society.

## Supplementary Material

Supplementary Data
