# Supplementary material for: Social and ecological factors influencing offspring survival in wild macaques
Source: Behav Ecol. 2014 Jun 17;25(5):1164–72. doi: 10.1093/beheco/aru099 (PMC4160111; doi:10.1093/beheco/aru099)
Supplement: Supplementary Data [file supp_25_5_1164__index.html]

Social and ecological factors influencing offspring survival in wild macaques — Social and ecological factors influencing offspring survival in wild macaques — Supplementary Data 

# Social and ecological factors influencing offspring survival in wild macaques

## Supplementary Data

Data files

**Files in this Data Supplement:**

- Supplementary Data - Supplementary Data
