# Supplementary material for: Social and ecological factors influencing offspring survival in wild macaques
Source: Behav Ecol. 2014 Jun 17;25(5):1164–72. doi: 10.1093/beheco/aru099 (PMC4160111; doi:10.1093/beheco/aru099)
Supplement: Supplementary Data [file supp_aru099_Supporting_Information.doc]

Supporting Information

|  | Study groups | | |
| --- | --- | --- | --- |
|  | R1 | R2 | PB |
| Study period (in months) | 58 | 58 | 36 |
| Number of adult males | 6-11 | 3-7 | 4-8 |
| Number of adult females | 18-24 | 13-21 | 15-18 |
| Number of study females | 23 | 20 | 17 |
| Number of conceptions | 39 | 37 | 23 |
| Number of fetal losses | 12 | 4 | 2 |
| Number of births | 31 | 38 | 9 |
| Number of infant losses | 5 | 12 | 0 |
| Number of male immigrations | 23 | 6 | 14 |
| Number of intergroup encounters | 459 | 125 | 142 |
| Number of alpha-male takeovers | 4 | 2 | 2 |
| Male hierarchy instability index* | 0.003 | 0.009 | 0.012 |

* (cf. Neumann et al. 2011)

**Table S1.** Demographic composition of the groups studied, social parameters and foetus/infant loss. The average of male hierarchy instability is calculated over all offspring intervals.

**Figure S2.** Distribution of conceptions (grey bars; monthly mean across all groups and years) and rainfall (black circles; monthly mean across all years with no offset) over the year. There is a significant negative correlation between monthly mean rainfall and monthly mean number of conceptions (rainfall: r=-0.769, P=0.003; Pearson’s Correlation Coefficient).

| Group | Infant | Female | Year | Wounds | Comment |
| --- | --- | --- | --- | --- | --- |
| R1 | US1 | US | 2006 | Yes | Dead body found with several cuts |
| R1 | GS2 | GS | 2009 | Unknown |  |
| R1 | JS2 | JS | 2009 | Unknown |  |
| R1 | WS1 | WS | 2010 | Yes | Wounds on forehead and face |
| R1 | ZS1 | ZS | 2010 | Unknown | Mother disappeared as well |
| R2 | LD1 | LD | 2006 | Yes |  |
| R2 | MD2 | MD | 2007 | Yes | Injured on hindquarters during an intergroup encounter |
| R2 | SD1 | SD | 2007 | Yes | Injured on head and one leg |
| R2 | UD1 | UD | 2007 | Unknown |  |
| R2 | UD2 | UD | 2008 | Unknown | Disappeared after an intergroup encounter |
| R2 | YD2 | YD | 2008 | Yes | Deep injury in the back |
| R2 | GD1 | GD | 2008 | Yes | Injured on head during intergroup encounter |
| R2 | TD2 | TD | 2009 | No | Big bruise on thorax, no open wounds |
| R2 | GD2 | GD | 2009 | Unknown | Disappeared with another infant |
| R2 | LD4 | LD | 2009 | Unknown | Mother disappeared at the same time |
| R2 | TD3 | TD | 2010 | Unknown |  |
| R2 | UD4 | UD | 2010 | Unknown |  |

**Table S3.** Group membership, identity of the dead/disappeared infant, year and circumstances of infant death/disappearance. The variable “Wounds” describes whether an open injury was observed on the infant’s dead body (“Yes/No”), while “Unknown” describes cases where the infant’s body was never found, thus presence of wounds is unknown.
